# Transcriptome Analysis Reveals the Effect of Low NaCl Concentration on Osmotic Stress and Type III Secretion System in *Vibrio parahaemolyticus*

**DOI:** 10.3390/ijms24032621

**Published:** 2023-01-30

**Authors:** Youkun Zhang, Xiaotong Tan, Mingzhu Li, Peng Liu, Xinan Jiao, Dan Gu

**Affiliations:** 1 Jiangsu Co-Innovation Center for Prevention and Control of Important Animal Infectious Diseases and Zoonoses, Yangzhou University, Yangzhou 225009, China; 2 Jiangsu Key Laboratory of Zoonosis, Yangzhou University, Yangzhou 225009, China; 3 Key Laboratory of Prevention and Control of Biological Hazard Factors (Animal Origin) for Agrifood Safety and Quality, Ministry of Agriculture of China, Yangzhou University, Yangzhou 225009, China

**Keywords:** *Vibrio parahaemolyticus*, RNA-seq, low NaCl, osmotic stress, T3SS

## Abstract

*Vibrio parahaemolyticus* is a moderately halophilic foodborne pathogen that is mainly distributed in marine and freshwater environments. The transition of *V. parahaemolyticus* between aquatic ecosystems and hosts is essential for infection. Both freshwater and host environments have low salinity. In this study, we sought to further investigate the effects of low salinity (0.5% NaCl) on the fitness and virulence of *V. parahaemolyticus*. We found that *V. parahaemolyticus* could survive in Luria–Bertani (LB) and M9 mediums with different NaCl concentrations, except for the M9 medium containing 9% NaCl. Our results further showed that *V. parahaemolyticus* cultured in M9 medium with 0.5% NaCl had a higher cell density than that cultured at other NaCl concentrations when it entered the stationary phase. Therefore, we compared the transcriptomes of *V. parahaemolyticus* wild type (WT) cultured in an M9 medium with 0.5% and 3% NaCl at the stationary phase using RNA-seq. A total of 658 genes were significantly differentially expressed in the M9 medium with 0.5% NaCl, including regulators, osmotic adaptive responses (compatible solute synthesis systems, transporters, and outer membrane proteins), and virulence factors (T3SS1 and T6SS1). Furthermore, a low salinity concentration in the M9 medium induced the expression of T3SS1 to mediate the cytotoxicity of *V. parahaemolyticus* to HeLa cells. Similarly, low salinity could also induce the secretion of the T3SS2 translocon protein VPA1361. These factors may result in the high pathogenicity of *V. parahaemolyticus* in low-salinity environments. Taken together, these results suggest that low salinity (0.5% NaCl) could affect gene expression to mediate fitness and virulence, which may contribute to the transition of *V. parahaemolyticus* between aquatic ecosystems and the host.

## 1. Introduction

*Vibrio parahaemolyticus* is distributed in estuarine and coastal environments and is considered a seafood-borne pathogen that can cause diarrhea if undercooked or raw seafood is consumed [1,2,3]. *V. parahaemolyticus* was first identified in a food poisoning outbreak in 1950 in Osaka, Japan [4]. Subsequently, this bacterium was identified in outbreaks and individual cases of gastroenteritis caused by seafood worldwide, particularly in coastal areas or country regions [5,6]. However, in recent years, *V. parahaemolyticus* has also been isolated from freshwater foods and environments [7,8,9]. In Malaysia, 69 (42%) *V. parahaemolyticus* isolates were isolated from freshwater fish samples from vet markets or supermarkets, and 15 out of the 69 isolates from freshwater fish were resistant to imipenem, which is the last-line antibiotic treatment for bacterial infections [10,11]. Similarly, 68 (16.2%) *V. parahaemolyticus* isolates could also be identified from freshwater food in fishing farms, retail markets, and restaurants in Zhejiang province, China, and three freshwater fish isolates belonged to the O3:K6 serotype, which is the pandemic serotype of *V. parahaemolyticus* [12]. Furthermore, two cases of foodborne disease caused by the small lobster were reported in Hubei province, China, confirming that freshwater food can carry *V. parahaemolyticus* and cause foodborne diseases [13]. Therefore, the virulence factors of *V. parahaemolyticus* cultured in different NaCl concentrations can be analyzed to elucidate the effects of salinity on virulence.

The main difference between freshwater and marine environments is the concentration of salinity, and pathogens can sense different NaCl concentrations to regulate the expression of adaptive responses and virulence factors to survive under osmotic stress [14,15,16]. Changes in osmolarity can trigger an efflux of water from cells that can cause a decrease in cytoplasmic volume and an increase in cytosolic ion concentration [17]. Adaptive stress response systems have been reported in *V. parahaemolyticus*, including compatible solute synthesis and transport systems, outer membrane proteins, and other salt-responsive genes (*exbB*, *exbD*, *groESL*, and *feoAB*) [18]. Ectoine, betaine, glycine betaine, and spermidine have been reported to be effective compatible solutes responsible for bacterial osmoadaptation [19,20,21]. In addition, outer membrane proteins can undergo remodeling under osmotic stress [18]. Low osmotic stress can repress OmpC expression and induce OmpF production to benefit bacteria by facilitating the influx of scarce nutrients in *Serratia marcescens* [22]. In *V. parahaemolyticus*, the expression of *ompW*, *ompN*, and *ompA2* was induced by 2% NaCl, whereas the expression of *ompU*, *ompA2*, and *VP1008* was repressed [18]. Furthermore, transcriptional factors can also be induced to regulate the expression of these genes under osmotic stress. The transcription and autophosphorylation levels of the two-component system EnvZ/OmpR were induced at high osmolality in *Salmonella* Typhimurium and *Escherichia coli* [14,23]. 

The main virulence factors of *V. parahaemolyticus* are the type III secretion system (T3SS) and type VI secretion system (T6SS), which can directly secrete effector proteins into host cells and cause disease [24]. *V. parahaemolyticus* contains two T3SSs (T3SS1 and T3SS2) and two T6SSs (T6SS1 and T6SS2), which can be induced or repressed under different culture conditions [24,25,26]. T6SS1 is mostly activated under high salinity and warm temperature conditions to enhance the environmental fitness of *V. parahaemolyticus*, and T6SS2 is activated during cold and warm temperatures in low salt conditions to increase the adhesion of *V. parahaemolyticus* to HeLa cells [25,27]. Almost all *V. parahaemolyticus* isolates contained T3SS1, which could be repressed in LBS, nitrite, and *exsD* expression, whereas induced in Dulbecco’s Modified Eagle Medium (DMEM) and *exsA* expression strains [28]. The T3SS1 contributes to cytotoxicity in vitro and mouse mortality in vivo [28]. T3SS2 is presumptively present in clinical isolates of *V. parahaemolyticus* and is induced by bile salts to cause gastroenteritis [29,30]. Motility and biofilm formation are also important virulence factors essential for the infection process and environmental fitness of *V. parahaemolyticus* [31,32,33]. However, few studies have focused on the effects of low salinity on *V. parahaemolyticus* virulence. Therefore, exploring the effects of salinity on *V. parahaemolyticus* virulence could provide comprehensive insights into its pathogenicity in both marine and freshwater environments.

The fitness of *V. parahaemolyticus* at low salinity and the effects of low salinity on its virulence remain unknown. In this study, we observed the growth curve of *V. parahaemolyticus* in LB and M9 medium containing different NaCl concentrations. Then, RNA-seq was performed to obtain the global transcriptome patterns of *V. parahaemolyticus* between M9 medium with 0.5% and 3% NaCl, and the transcriptional levels of compatible solute synthesis systems, transporters, and outer membrane proteins were verified by qRT-PCR. Furthermore, to the best of our knowledge, our results are the first to report that low NaCl concentrations induce the expression of T3SS1, which is responsible for *V. parahaemolyticus* cytotoxicity to HeLa cells and the secretion of the T3SS2 translocon protein VPA1361. Our findings provide new insights into the effects of low salinity on *V. parahaemolyticus* virulence.

## 2. Results

### 2.1. The Growth Curve of V. parahaemolyticus under Different NaCl Concentrations

**Figure 1:**
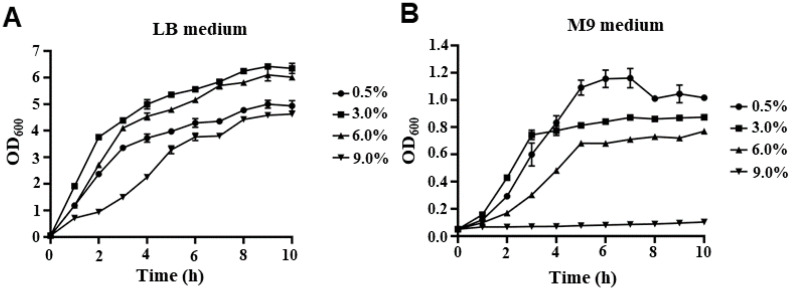
The growth curves of *V. parahaemolyticus*. *V. parahaemolyticus* strain RIMD2210633 was grown in LB (**A**) or M9 (**B**) mediums with 0.5%, 3%, 6%, and 9% NaCl. The bacteria were cultured at 37 °C by shaking at 180 rpm. The OD_600_ of each culture condition was monitored at the indicated times until the cultures reached the stationary phase.

To investigate the fitness of *V. parahaemolyticus* at different NaCl concentrations, the growth curves of the WT strain in LB and M9 medium with various NaCl concentrations are shown in Figure 1. In LB medium, *V. parahaemolyticus* can survive in 0.5% NaCl, as well as in salinity as high as 9% (Figure 1A). There was almost no lag phase of *V. parahaemolyticus* when cultured under salinity concentrations of 0.5%, 3%, and 6% NaCl, indicating that the strains quickly adapted to environmental changes and entered the exponential phase. However, the lag phase of cells cultured in LB medium with 9% NaCl was longer than that at other salinity concentrations. In addition, the growth rate under 9% NaCl was lower than that under other NaCl concentrations at the early exponential stage, whereas the growth rates under 3% and 6% NaCl were higher than those under 0.5% and 9% NaCl at the late exponential stage (Figure 1A). In the M9 medium, *V. parahaemolyticus* could survive under 0.5%, 3%, and 6% NaCl concentrations; however, it could not grow under 9% NaCl (Figure 1B). Furthermore, *V. parahaemolyticus* cultured in an M9 medium with 0.5% NaCl showed a higher cell density than at other NaCl concentrations when entering the stationary phase (Figure 1B), indicating that *V. parahaemolyticus* may have a stronger growth ability and occupy ecological niches under low salt concentrations.

### 2.2. Transcriptome Analysis of V. parahaemolyticus in M9 Medium with 0.5% or 3% NaCl

**Figure 2:**
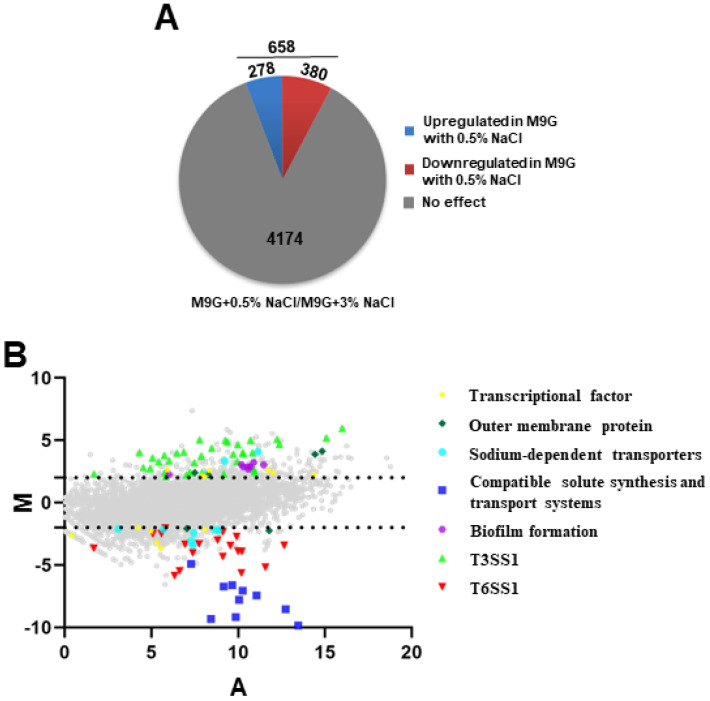
The effects of 0.5% NaCl on *V. parahaemolyticus* gene expression. (**A**) Pie charts show the differentially expressed genes under 0.5% NaCl in an M9 medium; (**B**) MA plots indicating the changes that were observed in gene expression at 0.5% NaCl compared to WT cultured in 3% NaCl. The genes associated with regulators, osmotic stress, and virulence are highlighted. X-axis means the average log2 value for transcript abundance under both conditions; Y-axis means the log2 values for the ratios of abundance for each transcript shown between WT cultured under 0.5% and 3% NaCl.

In this study, RNA-seq was used to identify the differentially expressed genes of *V. parahaemolyticus* under low-salinity conditions, which induced a stronger growth ability than the most suitable salinity concentration (3% NaCl). We compared the transcriptomes of *V. parahaemolyticus* WT grown in M9 medium with 0.5% and 3% NaCl and revealed that 658 genes significantly differed between 0.5% and 3% NaCl conditions (log_2_ fold change ≥ 2 or ≤−2, *p* < 0.05). As shown in Figure 2A and Table S3, 278 and 380 genes were upregulated and downregulated in 0.5% NaCl, respectively. Figure 2B shows the expression patterns of genes that are potentially associated with osmotic stress response and virulence, including transcriptional factors, outer membrane proteins, sodium-dependent transporters, compatible solute synthesis and transport systems, biofilm formation, T3SS1, and T6SS1. Furthermore, biofilm formation and T3SS1-associated genes were significantly upregulated, whereas the expression of T6SS1 was significantly downregulated in 0.5% NaCl compared with that in 3% NaCl. Taken together, these results indicate that low salinity could influence the expression of stress response and virulence genes in *V. parahaemolyticus*.

### 2.3. The Expression Levels of Osmotic Response Genes under Low NaCl Concentration

**Figure 3:**
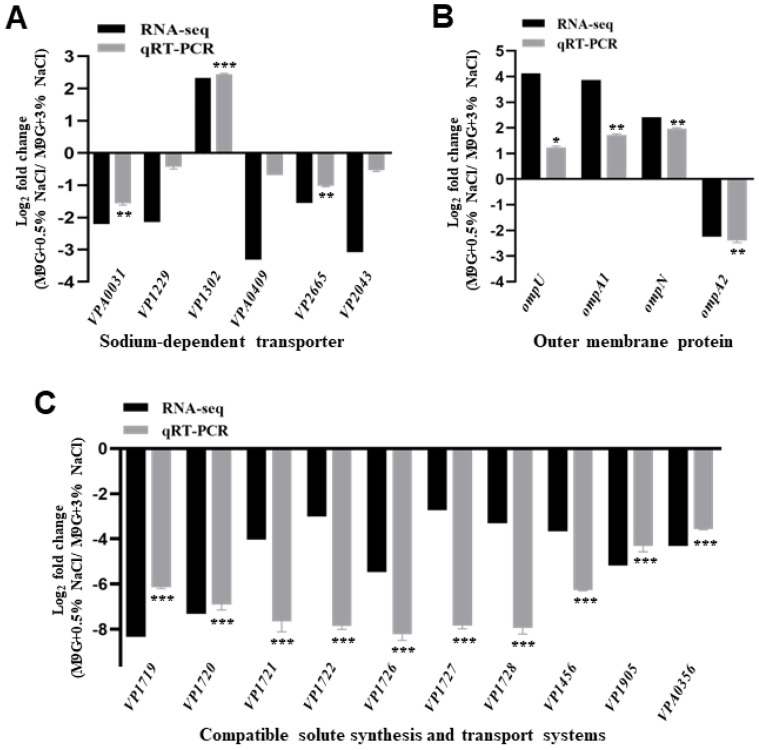
The transcript levels of putative osmoadaptation systems in *V. parahaemolyticus*. RNA-seq and qRT-PCR analysis for the transcription levels of the sodium-dependent transporter (**A**), outer membrane proteins (**B**), and compatible solute synthesis and transport systems (**C**). The data are presented as the mean ± standard deviation (SD) (n = 3). * *p* < 0.01, ** *p* < 0.001, *** *p* < 0.0001, Student’s *t*-test.

RNA-seq results revealed that the transcript levels of sodium-dependent transporters, outer membrane proteins, and compatible solute synthesis and transport systems were affected by low salinity, which may be responsible for osmotic stress. Our RNA-seq results showed that five transporter genes (*VPA0031*, *VP1229*, *VPA0409*, *VP2665*, and *VP2043*) were significantly downregulated, whereas only one gene (*VP1302*) was significantly upregulated in the WT strain when cultured in 0.5% NaCl compared with 3% NaCl (Figure 3A). Then, the qRT-PCR results confirmed that two genes (*VPA0031* and *VP2665*) were downregulated under low-salinity conditions, and the low-salinity condition could also induce a higher expression of *VP1302* (Figure 3A).

In the present study, RNA-seq and qRT-PCR results revealed that the expression of *ompU*, *ompA1*, and *ompN* was upregulated and that of *ompA2* was downregulated in 0.5% NaCl compared to that in 3% NaCl (Figure 3B). In addition, *VP1719-VP1722* (*ectABC*-*lysC*) is a major endogenous compatible solute synthesis in *V. parahaemolyticus*, which was significantly downregulated in 0.5% NaCl compared to 3% NaCl (Figure 3C). Furthermore, the transporters encoded by *VP1726-VP1728* (*proVWX*), *VP1456* (*opuD1*), *VP1905* (*opuD3*), and VPA0356 (*opuD4*) were significantly downregulated in 0.5% NaCl compared with 3% NaCl (Figure 3C). These results indicate that *V. parahaemolyticus* could regulate the expression of these genes in response to osmotic stress.

### 2.4. Low Concentration of NaCl Induces High Expression of T3SS1 and Contributes to the Cytotoxicity of V. parahaemolyticus

**Figure 4:**
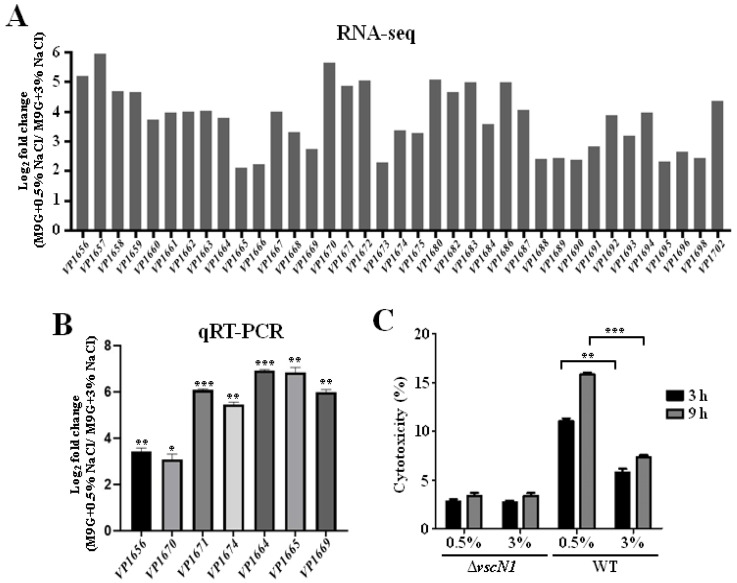
Low NaCl concentration induces the expression of T3SS1 and contributes to the cytotoxicity of *V. parahaemolyticus* to HeLa cells. (**A**) The transcription levels of T3SS1 identified by RNA-seq; (**B**) qRT-PCR analysis of the expression levels of T3SS1 genes in *V. parahaemolyticus* cultured in M9 medium with 0.5% NaCl compared to that in 3% NaCl. The data are presented as the mean ± SD (*n* = 3). * *p* < 0.01, ** *p* < 0.001, *** *p* < 0.0001, Student’s *t*-test; (**C**) cytotoxicity analysis of WT cultured in M9 medium with 0.5% or 3% NaCl to HeLa cells. The data are presented as the mean ± SD (*n* = 3). ** *p* < 0.001, *** *p* < 0.0001, Student’s *t*-test.

Our RNA-seq results also showed that the T3SS1 gene cluster was significantly upregulated in 0.5% NaCl compared with that in 3% NaCl. The differential expression levels of the T3SS1 genes are shown in Figure 4A. The qRT-PCR results confirmed that 0.5% NaCl induced the expression of T3SS1 genes (Figure 4B). A previous study revealed that T3SS1 could be induced in DMEM and contributed to the cytotoxicity of *V. parahaemolyticus* [28]. Therefore, the cytotoxicity of *V. parahaemolyticus* WT and Δ*vscN1* cultured in an M9 medium with 0.5% or 3% NaCl was also determined in this study. The results showed that the cytotoxicity of the WT strain cultured under 0.5% NaCl was significantly higher than that in 3% NaCl at 3 h or 9 h after the infection, and no difference was observed in the Δ*vscN1* strain which is a T3SS1-deficient mutant strain (Figure 4C) [34]. These results indicate that a low NaCl concentration can induce the expression of T3SS1 and contribute to the cytotoxicity of *V. parahaemolyticus* to HeLa cells. 

### 2.5. A Low NaCl Concentration Induces the Secretion of the T3SS2 Translocon Protein VPA1361

**Figure 5:**
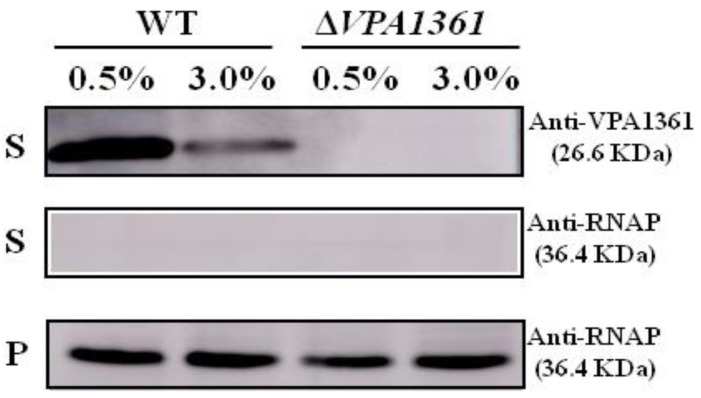
Low NaCl concentration induces the secretion of VPA1361. Western blotting analysis was performed to determine the secretion of VPA1361 in WT and Δ*VPA1361* in M9 medium with 0.5% or 3% NaCl. The supernatants (S) and cellular pellets (C) were collected and used for Western blotting analysis with VPA1361-specific antibodies. RNAP was used as the loading control of Western blotting.

Another important virulence factor of *V. parahaemolyticus* is T3SS2, which could be induced by bile salts and is responsible for enterotoxicity in vivo [34,35], and the VPA1361 has been characterized as the translocator of T3SS2 [36]. However, the bacteria used in the RNA-seq analysis were cultured in an M9 medium with different NaCl concentrations, which could not induce the expression and secretion of T3SS2. Thus, we detected the secretion of the T3SS2 translocon protein VPA1361 in the WT strain cultured under 0.5% or 3% NaCl following the induction of bile salts. In 0.5% NaCl, the secretion of VPA1361 was significantly higher than that in 3% NaCl (Figure 5). The *VPA1361* deletion mutant strain was used as the negative control. The RNAP antibody was used as the loading control. These results indicated that low salinity could induce the secretion of the T3SS2 translocon protein VPA1361 in *V. parahaemolyticus*.

## 3. Discussion

*V. parahaemolyticus* is a moderately halophilic, salt-requiring bacterium that can rapidly reproduce in a medium containing 3% NaCl, whereas it cannot survive under conditions with salt concentrations lower than 0.5% [32]. However, recent studies have shown that *V. parahaemolyticus* can be isolated from freshwater environments where the salt concentration is lower than 0.5% [37]. Therefore, we characterized the effects of low NaCl concentrations on the fitness and virulence of *V. parahaemolyticus* and identified differentially expressed genes under 0.5% NaCl in the M9 medium. We found that *V. parahaemolyticus* could survive in an LB medium containing the indicated concentrations of NaCl and quickly enter the exponential phase, except when cultured in 9% NaCl (Figure 1A). Previous studies have also shown that *V. parahaemolyticus* can quickly adapt to different pH and temperature conditions in the LB medium [38]. Likewise, these results indicate that *V. parahaemolyticus* could quickly adapt to environmental changes. However, few studies have focused on the survival ability of *V. parahaemolyticus* in an M9 medium, which is more similar to freshwater or seawater environments. Our results showed that *V. parahaemolyticus* could survive in the M9 medium with 0.5%, 3%, or 6% NaCl, but not at a NaCl concentration of 9% (Figure 1B). Interestingly, our results indicated that *V. parahaemolyticus* cultured in an M9 medium with 0.5% NaCl had a higher cell density than that in the optimum growth NaCl concentration (3%) in the stationary phase (Figure 1B), which was the opposite in the LB medium. The LB medium was a nutritionally complex medium, and all *V. parahaemolyticus* strains were grown aerobically in LB, whereas the M9 medium was a simple medium consisting of salts to which amino acids and carbon sources can be added. Furthermore, the OD_600_ value of WT cultured at LB was higher than that in the M9 medium at the stationary phase. Furthermore, the growth ability of *Vibrio brasiliensis* cultured under the condition of low NaCl concentration was lower than that cultured under the optimum condition [39]. It is important to explore the biological significance of *V. parahaemolyticus*, which has a stronger growth advantage under conditions of low NaCl concentration. Thus, in this study, we investigated the effects of 0.5% NaCl on the virulence and differentially expressed genes in *V. parahaemolyticus*. 

The ability of microorganisms to adapt to environmental changes or stress is essential for bacteria to occupy niches and survive under different conditions. Osmolarity is a key factor that changes between marine and freshwater environments, and our RNA-seq and qRT-PCR results indicated that the sodium-dependent transporters, compatible solute synthesis, and transport systems, and outer membrane proteins were significantly influenced by the NaCl concentration in the M9 medium. Three Na^+^/H^+^ antiporters (NhaA, NhaB, and NhaD) have been reported to contribute to cytoplasmic Na^+^ circulation in *V. parahaemolyticus*, and the binding of Na^+^ to the transporter may induce changes in its stability [40,41]. Our results also indicated that five sodium-dependent transporters were downregulated in 0.5% NaCl compared to 3% NaCl, and only one transporter (VP1302) was induced under low-salinity conditions (Figure 3A). Notably, the expression of the VP1302 transporter was significantly increased under the 0.5% NaCl condition, whereas the other transporters were downregulated under low-salinity conditions. VP1302 is an L-cystine symporter responsible for the transport of L-cystine into the cell; thus, it can be speculated that cystine may be a compatible solute for *V. parahaemolyticus* osmoadaptation. The compatible solute synthesis systems (*ectABC*/*lysC*) and three compatible solute transporters (*proU*, *opuD1*, and *potA2B2C2*) were upregulated under 2% NaCl in *V. parahaemolyticus* [18]. Our RNA-seq results also showed that *ectABC*/*lysC*, *proU*, and *opuD1* were highly expressed in 3% NaCl (Figure 3C), but the *potA2B2C2* genes were not affected by NaCl concentration, which may be due to the different concentrations of NaCl or the culture medium. Additionally, we identified two other compatible solute transporters (*opuD3* and *opuD4*) that were significantly induced by 3% NaCl (Figure 3C). Furthermore, our results showed that outer membrane proteins were affected by NaCl concentration, which was confirmed in previous studies [42,43]. Previous studies have shown that outer membrane proteins are important for membrane stability, pathogenicity, and bacterial survival under environmental stress in *Acinetobacter baumannii*, *Helicobacter pylori*, *E. coli*, and *Vibrio cholerae* [44,45,46,47]. These results demonstrated that *V. parahaemolyticus* could regulate the expression of compatible solute synthesis systems, transporters, and outer membrane protein response to osmotic stress.

The ability of bacteria to transition between aquatic systems and the human host is essential for the infection process of pathogens, and this ability requires the regulation of adaptive responses and virulence factor expression [14,48]. The *V. cholerae* two-component system EnvZ/OmpR can sense osmolarity and acidic pH signals to regulate the expression of virulence factor [14]. *V. parahaemolyticus* contains two T3SSs that could be induced or inhibited by different signals through the regulators to mediate cytotoxicity and gastroenteritis, respectively [26,35]. The *V. parahaemolyticus* two-component system VbrK/VbrR can sense NO_2_^-^ to inhibit the expression of T3SS1, and VtrA/VtrB can sense bile salts to induce the expression of T3SS2 [26,49]. Furthermore, our results showed that 0.5% NaCl induced the expression of T3SS1 to increase the cytotoxicity of *V. parahaemolyticus* to HeLa cells (Figure 4), and the secretion of the T3SS2 translocon protein VPA1361 was increased by 0.5% NaCl in the M9 medium (Figure 5). However, in our RNA-seq results, the VtrA/VtrB and VbrK/VbrR were not affected by low salinity. Thus, we speculate that 0.5% NaCl may influence other regulatory genes to regulate the expression of T3SS1 and T3SS2. 

In conclusion, we explored the effects of 0.5% NaCl on the fitness and virulence of *V. parahaemolyticus*. Our results indicate that *V. parahaemolyticus* can survive under different NaCl concentrations, except for an M9 medium containing 9% NaCl. The expression of compatible solute synthesis systems, transporters, and outer membrane proteins was affected in an M9 medium with 0.5% NaCl, which is responsible for osmotic stress. Furthermore, the *V. parahaemolyticus* virulence factors T3SS1 and T3SS2 were induced under 0.5% NaCl conditions. These findings indicate that *V. parahaemolyticus* can sense low-salinity signals to regulate the expression of adaptive responses and virulence factors, and then fitness under low-salinity conditions.

## 4. Materials and Methods

### 4.1. Bacterial Strain and Culture Conditions 

The strains and plasmids used in the present study are listed in Table S1. All *V. parahaemolyticus* strains were cultured at 37 °C in LB or M9 mediums with the indicated NaCl concentrations. The following antibiotics were added when required: carbenicillin (Carb, 100 μg/mL) and chloramphenicol (Cm, 40 μg/mL). In addition, bile salts (0.04%, *w*/*v*) were used to induce the expression of VPA1361 in the WT and Δ*VPA1361* strains.

### 4.2. Growth Curve 

The growth curves of *V. parahaemolyticus* WT were detected in LB and M9 mediums containing 0.5%, 3.0%, 6.0%, and 9.0% NaCl. One WT colony was collected and cultured in an LB medium with 3.0% NaCl at 37 °C. Overnight cultured bacteria were diluted into 50 mL fresh LB or M9 mediums with different NaCl concentrations, and the initial OD_600_ value was adjusted to 0.05. Bacteria cultures (200 μL) were used to detect the absorbance at OD_600_ using a microplate reader. The experiments were repeated three times. 

### 4.3. RNA-Seq Analysis 

The *V. parahaemolyticus* RIMD2210633 strain was cultured in LB broth at 37 °C for 12 h. The cultures were then diluted into a fresh M9 medium with 0.5% and 3.0% NaCl and cultured at 37 °C for 5–6 h until the stationary phase was reached. Total RNA was extracted using the RNeasy Plus Mini Kit (Qiagen, Hilden, Germany), the concentration of total RNA was determined by a Qubit 2.0 Fluorometer (Thermo Fisher Scientific, Waltham, MA, USA), and the integrity was determined by electrophoresis. Next, tRNA was removed by Ribo-Zero-rRNA removal kits (Thermo Fisher Scientific, MA, USA) and the first-strand cDNA was synthesized. This was followed by second-strand cDNA synthesis, end repair, 3′ end adenylation, and adapter ligation. Finally, the library was amplified via PCR with 10 cycles, and the fragments ligated to the sequencing adapters were enrichment. Three biological replicates were sequenced using Illumina HiSeq (GENEWIZ, Suzhou, China), and statistical analyses were performed as described previously [50].

### 4.4. qRT-PCR

Bacterial culture conditions and total RNA extracts were performed as the RNA-seq analysis. RNase-free DNase I was used to remove genomic DNA, and 1 μg RNA was used to generate cDNA using Hiscript III RT Super mix (Vazyme, Nanjing, China). The specific primers used for qRT-PCR are listed in Table S2. The reaction mixtures were run on an ABI PRISM 7500 Real-Time PCR System (Applied Biosystems, Foster City, CA, USA) using Universal SYBR qPCR Master Mix (Vazyme, Nanjing, China). Transcript levels of the indicated genes were normalized to *gyrB* using the 2^−ΔΔCt^ method [51]. Three independent experiments were performed.

### 4.5. Cytotoxicity Analysis

The overnight cultured *V. parahaemolyticus* RIMD2210633 strain was diluted into a fresh M9 medium with 0.5% or 3.0% NaCl, and then cultured at 37 °C for 5–6 h by shaking. The bacteria were collected and resuspended in Dulbecco’s Modified Eagle Medium (DMEM). The bacterial suspensions were inoculated with HeLa cells at an MOI of 100 CFU/cell. After 3 h and 9 h of exposure to WT, the release of lactate dehydrogenase (LDH) in the supernatant was measured using an LDH Cytotoxicity Assay Kit according to the manufacturer’s instructions (Beyotime, Shanghai, China).

### 4.6. Western Blotting

For VPA1361 immunoblotting analysis, the overnight cultured WT was diluted into an M9 medium with 0.5% or 3% NaCl and cultured at 37 °C for 5–6 h. Next, 0.04% bile salt was added to induce T3SS2 expression. The bacterial cultures were adjusted based on the OD_600_ value of the WT and Δ*VPA1361* strains, then the cell pellets and supernatant were collected. The supernatant protein was concentrated using protein filter columns Ultracel and Regenerated Cellulose (Millipore, Billerica, MA, USA), and the cell pellets were suspended in PBS. Loading buffer was added to each sample, and the mixture was boiled for 10 min. A 20 μL aliquot of each normalized sample was then separated on a 12% polyacrylamide gel and transferred to PVDF membranes (Millipore, Billerica, MA, USA). After blocking in 10% skim milk for 2 h, the PVDF membranes were incubated with the VPA1361 antibody or RNAP antibody at a 1:1000 dilution and then incubated with goat anti-rabbit IgG (Beyotime, Shanghai, China) at a 1:2000 dilution. Finally, the blots were visualized using ECL reagent.

## Data Availability

Raw sequencing reads of RNA-seq were deposited in the European Nucleotide Archive database under accession number PRJEB57782.

## Supplementary Materials

The following supporting information can be downloaded at: https://www.mdpi.com/article/10.3390/ijms24032621/s1. References [34,52,53,54,55] are cited in supplementary material file.
