# Supplementary material for: Transcriptome Analysis Reveals the Effect of Low NaCl Concentration on Osmotic Stress and Type III Secretion System in Vibrio parahaemolyticus
Source: Int J Mol Sci. 2023 Jan 30;24(3):2621. doi: 10.3390/ijms24032621 (PMC9916905; doi:10.3390/ijms24032621)
Supplement: Supplementary file 1 [file ijms-24-02621-s001.zip › ijms-2086572-supplementary.pdf]

Table S1. The Bacterial strains and plasmids used in this study

Strains or plasmids	Relevant characteristics	Reference
E. coli		
DH5α λ*pir*	Host for π requiring plasmids	[34]
SM10 λ*pir*	Host for π requiring plasmids, conjugal donor	[52]
V. Parahaemolyticus		
RIMD2210633	Wild type, O3:K6 clinical isolate, Carb^r	[53]
Δ*VPA1361*	RIMD2210633, in-frame deletion in *VPA1361*, Carb^r	This study
Δ*vscN1*	RIMD2210633, in-frame deletion in *vscN1*, Carb^r	[54]
**Plasmids**		
pDM4	Suicide vector, λ*pir* dependent, R6K, *SacBR*, Cm^r	[55]
pDM4::Δ*VPA1361*	Up and downstream of *VPA1361* insert into pDM4, Cm^r	This study

Table S2. Primers used in this study

Primer name	Primer sequence (5' to 3')	Target
VPA1361 up-F	GAGCGGATAACAATTTGTGGAATCCCGGGATTGGTGGTATGGTAGCCAGTTTGGT	For *VPA1361* mutant
VPA1361 up-R	GTAACCAAACTCATAATTTTCTCCTTACATATGAAT	For *VPA1361* mutant
VPA1361 down-F	GGAGAAAATTATGAGTTTGGTTACTAACCGTTAATTAG	For *VPA1361* mutant
VPA1361 down-R	AGCGGAGTGTATATCAAGCTTATCGATACCAAAGCGTCGACACAGAACCAACATG	For *VPA1361* mutant
VPA1361 out-F	TCGCAATTGGAAGAACAGAAGAAGG	For *VPA1361* mutant
VPA1361 out-R	GATCCAGCGTTGTCTGTTTCAAGTT	For *VPA1361* mutant
VPA1361 in-F	CTGTAAAAGGCACCGCTATTGATAC	For *VPA1361* mutant
VPA1361 in-R	TAACCATCTAGCATTGCGTCAACTT	For *VPA1361* mutant
pDM4-F	CAGCAACTTAAATAGCCTCTAAGGT	For *VPA1361* mutant
pDM4-R	GGTGCTCCAGTGGCTTCTGTTTCTA	For *VPA1361* mutant
VP1656-F	CTGTAGCACCCGCTAACACA	For qRT-PCR
VP1656-R	GCGGTTTCTCAATCGCTGTC	For qRT-PCR
VP1670-F	CTCGCCTCTCATCCTGTCAC	For qRT-PCR
VP1670-R	GGTTGAGTAGGTTGTGGGGG	For qRT-PCR
VP1671-F	GCCCTTCCTGTCTGGCTAAG	For qRT-PCR
VP1671-R	AGTTGCTGGTGGTCTGTGAG	For qRT-PCR
VP1674-F	TTCTCAATCCACCCCAACGG	For qRT-PCR
VP1674-R	CGATGGCACTTTTGATGGGC	For qRT-PCR
VP1664-F	ATTCCTGTGCGAGTTGCTGA	For qRT-PCR
VP1664-R	GCGTTAGCGAAGCCAAAACT	For qRT-PCR
VP1665-F	TTTGGCGGAAAACACCAAGG	For qRT-PCR
VP1665-R	AGCGGCACATTGCACATAGA	For qRT-PCR
VP1669-F	ATCACGTTTGGCGTCAGGAA	For qRT-PCR
VP1669-R	GCGATTGTTCCGCGATTTCT	For qRT-PCR
VPA0031-F	AGGAGGCGCGAAGGTTTTTA	For qRT-PCR
VPA0031-R	AAACCAAAAGTGGCCGATGC	For qRT-PCR
VP1229-F	ACTCGATGTCGCAGAAGGAC	For qRT-PCR
VP1229-R	CATGTTGTAGCTTTCGCGGG	For qRT-PCR
VP1302-F	TTCAGTTAGCCCTTCCGCTG	For qRT-PCR
VP1302-R	TTGGCTTGGCTCTTCAGCTT	For qRT-PCR
VPA0409-F	TGCGTGAATCGTACCTGGC	For qRT-PCR
VPA0409-R	GCTGTGTATCTTTCTTCCACCG	For qRT-PCR
VP2665-F	ACGAGTCTTCCTGAGCTTGC	For qRT-PCR
VP2665-R	AACAGAATCGCCCCTTCGAG	For qRT-PCR
VP2869-F	GGTGCGACGTTTGTCCTCTA	For qRT-PCR
VP2869-R	CGTAGGTAAGGGGCAAGCAA	For qRT-PCR
VP2337-F	TAACCATTGAGTCGCCGCTT	For qRT-PCR
VP2337-R	ATGGTGGTGGAGCCTTCTTG	For qRT-PCR
VP2043-F	TACATTCTTGGCCCGCTGTT	For qRT-PCR

VP2043-R	AAAGCCAGTGCCGATTACCA	For qRT-PCR
VP1635-F	TGGCTGTGGCGAATACATCA	For qRT-PCR
VP1635-R	CGTTTTTCGCTCGTCGTTCA	For qRT-PCR
VP2467-F	GCAGCAACCTAGACACTCGT	For qRT-PCR
VP2467-R	AGTAGCCGTCTTGACCGTTG	For qRT-PCR
VPA1186-F	CGACGGCAATTTAACGGCTT	For qRT-PCR
VPA1186-R	ACCGAAGAAGTTTGCGTCCA	For qRT-PCR
VPA0248-F	GGCCTAGAAAGCGCAGAAGT	For qRT-PCR
VPA0248-R	TACTTCCACACGACGGTTCG	For qRT-PCR
VPA0527-F	CTCTTACCGCAACGCAATCG	For qRT-PCR
VPA0527-R	ACCGTAAGAACCGTATGCCG	For qRT-PCR
VPA0316-F	CTAACTGGTGACGAAGGCCA	For qRT-PCR
VPA0316-R	GAACGCGCTGATTTCTGCTT	For qRT-PCR
VPA0362-F	GTAGCGTCCAATCACCACCA	For qRT-PCR
VPA0362-R	AGAAACCGAATCCACGCTGT	For qRT-PCR
VP1719-F	TGTGGCAGATAGTGACGCAG	For qRT-PCR
VP1719-R	AAGACGCCCTAGCGTATGTG	For qRT-PCR
VP1720-F	GGATTGAAAACGCACGCCAT	For qRT-PCR
VP1720-R	TGAAGGAACGGAAACGCACA	For qRT-PCR
VP1721-F	AACAGCGCCAAACCATCAAC	For qRT-PCR
VP1721-R	GTTCAATTCACCGGCCCAAC	For qRT-PCR
VP1722-F	TAACCCGAGATGAAGCCAGC	For qRT-PCR
VP1722-R	ATCACATCAGCACCTTGGGT	For qRT-PCR
VP1726-F	TAATGTCGCCAGCGTGCTAA	For qRT-PCR
VP1726-R	GAGCGATTTTCCTTGTGGGC	For qRT-PCR
VP1727-F	CAAGATGCCGTTCTCCCACT	For qRT-PCR
VP1727-R	AGAAAACTGCCACGCGAGTA	For qRT-PCR
VP1728-F	GGCGTACTTGTTCCGGGTAA	For qRT-PCR
VP1728-R	TTACGTGCGCTTCGATGTCT	For qRT-PCR
VP1456-F	TGGCTGTCAATGCTGTTTGC	For qRT-PCR
VP1456-R	CAACGCAACAAGGGCGTAAA	For qRT-PCR
VP1905-F	GCCAGACCACCAAATACCGA	For qRT-PCR
VP1905-R	TAACCCGTTTGGTCGTACCG	For qRT-PCR
VPA0356-F	TACCCTGTACGACCAAACGC	For qRT-PCR
VPA0356-R	GCCTCGAACTCACCAATCCA	For qRT-PCR
VP1273-F	CGACACTTTTTCACGCAGCA	For qRT-PCR
VP1273-R	TGCTCAAGTAACCTCTGCGG	For qRT-PCR

Table S3. The differentially expressed genes of *V. parahaemolyticus* WT cultured in 0.5% NaCl compared to 3% NaCl

Locus_tag	Product	Log2foldchange (0.5% NaCl/3% NaCl)	P-value
VP0008	amino acid ABC transporter substrate-binding protein	2.64	2.80E-29
VP0018	16 kDa heat shock protein A	-2.34	0.000337784
VP0020	periplasmic alpha-amylase	2.75	1.42E-58
VP0026	hypothetical protein	-2.39	3.43E-08
VP0035	ketol-acid reductoisomerase	2.46	1.47E-30
VP0048	peptide ABC transporter periplasmic peptide-binding protein	3.32	6.65E-66
VP0049	peptide ABC transporter permease	2.05	1.23E-28
VP0055	RNA polymerase ECF-type sigma factor	2.61	9.87E-18
VP0063	thermoresistant gluconokinase	-2.08	0.011797317
VP0069	hypothetical protein	2.35	2.65E-38
VP0074	hypothetical protein	2.55	1.48E-33
VP0079	hypothetical protein	-2.91	8.49E-16
VP0082	transmembrane protein	-2.69	0.000174917
VP0118	nitrogen regulation protein	2.65	7.31E-35
VP0119	nitrogen regulation protein NR(II)	5.27	1.18E-185
VP0120	hypothetical protein	2.17	1.88E-10
VP0121	glutamine synthetase	4.54	1.26E-86
VP0131	hypothetical protein	-2.06	0.000984576
VP0156	xanthine/uracil permease family protein	4.24	1.42E-58
VP0175	hypothetical protein	-2.22	4.04E-07
VP0181	bifunctional phosphopantothenoylcysteine decarboxylase/phosphopantothenate synthase	2.25	9.83E-52
VP0218	hypothetical protein	-2.15	3.93E-15
VP0225	capsular polysaccharide biosynthesis protein CapF	-2.35	6.42E-06
VP0226	rhamnosyl transferase	-3.88	3.50E-09
VP0227	hypothetical protein	-2.59	0.000539753
VP0228	integral membrane protein	-3.37	0.00085569
VP0229	dTDP-4-dehydrorhamnose 3%2C5-epimerase	-2.15	5.10E-06
VP0230	glycosyltransferase	-2.05	4.05E-14
VP0238	hypothetical protein	2.27	3.72E-11
VP0239	triosephosphate isomerase	3.08	5.58E-60
VP0243	hypothetical protein	-2.20	2.21E-21
VP0244	fructose 1%2C6-bisphosphatase II	2.47	3.32E-38
VP0287	ElaA protein	2.40	2.56E-10
VP0292	sulfate adenylyltransferase	2.89	1.05E-21
VP0293	sulfate adenylyltransferase	3.03	7.33E-24
VP0305	hypothetical protein	-2.51	0.001067874
VP0311	inorganic pyrophosphatase	3.15	2.16E-55
VP0319	hypothetical protein	2.82	3.55E-74
VP0323	hypothetical protein	2.00	3.15E-29

VP0332	hypothetical protein	2.15	2.40E-09
VP0343	isopropylmalate isomerase large subunit	2.30	7.48E-33
VP0348	hypothetical protein	-2.73	0.031131516
VP0350	leucine transcriptional activator	2.24	9.74E-37
VP0355	LuxZ	-3.64	3.95E-05
VP0356	pyruvate kinase	3.41	1.20E-99
VP0359	glucosamine--fructose-6-phosphate aminotransferase	3.06	3.82E-43
VP0371	hypothetical protein	-3.09	0.011817712
VP0375	lipoprotein	-2.02	1.22E-06
VP0378	hypothetical protein	2.07	6.39E-20
VP0379	ABC transporter substrate binding protein	2.10	2.78E-16
VP0384	hypothetical protein	-2.36	0.041339058
VP0396	hypothetical protein	-2.80	0.026060595
VP0399	transcriptional regulator	-2.62	0.039991954
VP0401	hypothetical protein	-2.27	6.45E-10
VP0496	threonine synthase	2.53	1.24E-54
VP0525	hypothetical protein	3.54	3.65E-96
VP0526	NptA protein	-4.44	8.95E-06
VP0540	carbon starvation protein A	2.74	6.74E-36
VP0542	hypothetical protein	-2.21	2.55E-06
VP0546	phospho-2-dehydro-3-deoxyheptonate aldolase	-3.78	3.25E-88
VP0547	bifunctional chorismate mutase/prephenate dehydrogenase	-3.06	2.05E-99
VP0551	ABC transporter ATP-binding protein	2.41	3.30E-36
VP0553	Trp operon repressor	-2.06	0.000707365
VP0562	hypothetical protein	-3.48	1.54E-21
VP0563	hypothetical protein	-3.24	4.39E-09
VP0583	malate synthase	2.42	1.14E-49
VP0584	isocitrate lyase	2.41	2.50E-42
VP0598	HesB family protein	-2.23	2.16E-26
VP0623	D-amino acid dehydrogenase small subunit	-2.26	4.57E-16
VP0626	hypothetical protein	-2.52	2.15E-05
VP0629	homocysteine synthase	2.30	4.29E-25
VP0633	integral membrane protein	-3.34	1.34E-11
VP0666	phosphoribosylformylglycinamidine synthase	2.44	3.58E-29
VP0667	hypothetical protein	-3.21	2.08E-12
VP0668	hypothetical protein	-3.29	3.26E-06
VP0669	hypothetical protein	2.37	2.54E-41
VP0670	hypothetical protein	-2.35	1.15E-10
VP0672	hypothetical protein	2.13	3.61E-20
VP0696	minor nuclease C1B isoform	-3.60	3.83E-05
VP0706	DL-methionine transporter ATP-binding subunit	3.20	6.13E-52
VP0715	serine hydroxymethyltransferase	3.94	1.76E-85
VP0718	hypothetical protein	-3.18	1.16E-05
VP0752	hypothetical protein	-2.96	3.28E-28

VP0756	hypothetical protein	-2.64	0.024614613
VP0768	hypothetical protein	2.49	9.01E-33
VP0781	flagellar basal body rod protein FlgG	2.94	5.43E-51
VP0782	flagellar basal body L-ring protein	2.66	5.05E-43
VP0788	flagellin	2.32	7.14E-35
VP0790	flagellin	3.61	3.91E-66
VP0794	phosphoenolpyruvate-protein phosphotransferase	2.25	2.62E-37
VP0796	hypothetical protein	-2.51	6.45E-05
VP0802	hypothetical protein	-3.01	9.98E-12
VP0807	hypothetical protein	-3.42	0.001111946
VP0821	heat shock protein 90	-2.02	0.005618581
VP0826	asparagine synthetase B	4.26	1.44E-69
VP0830	hypothetical protein	-4.46	2.88E-06
VP0855	hypothetical protein	-3.08	0.012211927
VP0864	formyltetrahydrofolate deformylase	3.00	3.42E-66
VP0872	cell division topological specificity factor MinE	2.05	3.33E-33
VP0879	bifunctional 2C10-methylene-tetrahydrofolate dehydrogenase methylene tetrahydrofolate cyclohydrolase	2.44	7.43E-51
VP0895	acyl carrier protein	-3.87	0.000776202
VP0905	KtrB protein	-2.49	4.01E-13
VP0907	hypothetical protein	-2.79	4.06E-29
VP0910	C4-dicarboxylate-binding periplasmic protein	-2.24	4.91E-07
VP0912	C4-dicarboxylate transport protein	-2.17	0.002685492
VP0938	hypothetical protein	-2.64	3.92E-12
VP0942	fimbrial protein	-4.15	6.86E-15
VP0950	lipoprotein-like protein	-3.50	1.04E-07
VP0951	methylated-DNA-protein-cysteine methyltransferase-like protein	-2.54	0.00371212
VP0955	ferredoxin	-2.38	1.10E-06
VP0969	hypothetical protein	2.33	6.44E-33
VP0990	hypothetical protein	2.01	4.33E-32
VP0994	formate acetyltransferase	3.58	1.87E-66
VP0999	amino acid ABC transporter substrate-binding protein	2.83	7.08E-43
VP1013	ATP-dependent Clp protease adaptor protein ClpS	-2.77	1.59E-12
VP1016	translation initiation factor IF-1	-2.37	5.44E-06
VP1021	hypothetical protein	-3.43	1.54E-05
VP1029	hypothetical protein	-2.58	4.33E-06
VP1038	hypothetical protein	-2.65	0.015504005
VP1050	hypothetical protein	-2.14	0.013499951
VP1058	TolR membrane protein	-2.53	6.39E-12
VP1073	immunity repressor protein	-2.88	0.021329999
VP1074	hypothetical protein	-2.51	3.80E-13
VP1076	hypothetical protein	-3.29	5.62E-35
VP1095	hypothetical protein	-2.68	6.96E-09

VP1096	tryptophanyl-tRNA synthetase II	-2.54	2.18E-25
VP1128	adenylosuccinate lyase	2.75	1.88E-55
VP1134	hypothetical protein	3.34	9.00E-46
VP1157	formate transporter 1	-3.23	6.08E-12
VP1167	peptide ABC transporter ATP-binding protein	2.29	3.11E-42
VP1174	phage shock protein B	-2.85	8.04E-12
VP1175	phage shock protein C	-3.12	3.68E-18
VP1185	chemotaxis transducer	-2.74	4.86E-12
VP1188	ferredoxin oxidoreductase protein	2.90	5.90E-24
VP1189	hydroxylamine reductase	4.59	1.39E-152
VP1190	anaerobic nitric oxide reductase transcription regulator	2.09	1.03E-18
VP1196	ABC transporter solute-binding protein	2.17	1.96E-18
VP1203	heat shock protein HslJ	-2.02	7.77E-06
VP1208	amino group acetyl transferase	-3.46	0.003770392
VP1229	Na+/H+-antiporter protein	-2.15	6.29E-11
VP1241	hypothetical protein	-3.97	6.93E-69
VP1247	3-phosphoserine/phosphohydroxythreonine aminotransferase	2.78	1.25E-64
VP1254	hypothetical protein	-2.51	4.16E-10
VP1263	phosphoribosylaminoimidazole-succinocarboxamide synthase	4.61	1.17E-81
VP1264	hypothetical protein	-2.66	8.34E-09
VP1268	hypothetical protein	-2.02	7.75E-09
VP1289	GGDEF family protein	-2.73	0.000193196
VP1297	phosphoribosylglycinamide formyltransferase 2	3.74	3.68E-72
VP1300	hypothetical protein	-3.08	5.60E-05
VP1302	sodium/dicarboxylate symporter	2.33	4.46E-15
VP1311	vtamin B12-transporter permease	-2.66	3.03E-18
VP1329	fatty aldehyde dehydrogenase	2.09	2.08E-07
VP1348	M20A family peptidase	3.71	2.10E-87
VP1349	4-hydroxyphenylpyruvate dioxygenase	2.09	6.93E-14
VP1354	hypothetical protein	-2.70	0.032924634
VP1368	hypothetical protein	-2.72	0.01360393
VP1369	hypothetical protein	-2.06	0.000202311
VP1372	hypothetical protein	-2.40	3.92E-06
VP1374	transporter	2.80	2.91E-39
VP1375	sensor histidine kinase	2.08	6.02E-16
VP1376	chemotaxis protein CheY	2.17	7.25E-27
VP1386	hypothetical protein	-3.94	6.04E-45
VP1391	transcriptional regulator	-3.44	4.13E-56
VP1393	BfdA protein	-3.89	2.29E-66
VP1394	VgrG protein	-2.34	2.20E-31
VP1395	hypothetical protein	-2.50	6.92E-09
VP1396	hypothetical protein	-2.49	8.21E-12
VP1399	hypothetical protein	-2.04	5.23E-10
VP1400	hypothetical protein	-5.64	3.16E-121

VP1401	hypothetical protein	-5.18	2.06E-145
VP1402	hypothetical protein	-4.32	2.96E-87
VP1403	hypothetical protein	-3.42	4.03E-89
VP1404	hypothetical protein	-5.87	3.87E-24
VP1405	hypothetical protein	-3.91	1.01E-99
VP1406	hypothetical protein	-3.35	6.49E-27
VP1410	hypothetical protein	-4.03	6.85E-43
VP1411	hypothetical protein	-3.00	1.27E-42
VP1412	hypothetical protein	-5.47	3.50E-29
VP1413	hypothetical protein	-2.73	4.92E-53
VP1414	hypothetical protein	-3.31	1.66E-49
VP1418	hypothetical protein	-3.66	0.001690505
VP1421	hypothetical protein	-3.37	0.001218218
VP1426	tyrosine-specific transport protein	-2.18	1.16E-32
VP1434	V10 pilin	2.89	4.73E-17
VP1441	GGDEF family protein	-2.03	1.01E-16
VP1448	anaerobic dimethyl sulfoxide reductase subunit B	2.44	1.45E-53
VP1449	anaerobic dimethyl sulfoxide reductase subunit C	2.13	1.73E-32
VP1452	azoreductase	2.65	9.09E-24
VP1456	BCCT family transporter	-6.72	1.53E-82
VP1458	hypothetical protein	4.54	5.60E-37
VP1467	galactosyltransferase	2.34	3.27E-07
VP1470	hypothetical protein	-3.18	0.009011558
VP1481	hypothetical protein	-2.12	1.60E-17
VP1483	hypothetical protein	-2.69	6.83E-17
VP1484	hypothetical protein	-2.84	1.01E-07
VP1490	hypothetical protein	-3.22	0.001929315
VP1491	hypothetical protein	-3.89	0.000682809
VP1493	hypothetical protein	-2.21	0.000361405
VP1495	hypothetical protein	-2.63	0.038826559
VP1501	hypothetical protein	2.26	1.29E-45
VP1525	spermidine/putrescine ABC transporter periplasmic spermidine/putrescine-binding protein	2.00	1.13E-31
VP1542	cytochrome c oxidase subunit CcoQ	-2.19	1.43E-08
VP1545	hypothetical protein	-2.25	0.008032621
VP1566	structural protein P5	2.37	0.007288728
VP1593	ribosome modulation factor	-3.00	1.76E-21
VP1601	dihydroorotate dehydrogenase 2	2.20	2.29E-31
VP1620	amino acid ABC transporter substrate-binding protein	2.04	9.08E-23
VP1625	hypothetical protein	2.79	3.26E-28
VP1626	sulfite reductase%2C gamma subunit-like protein	-3.43	0.000683636
VP1634	agglutination protein	2.78	7.49E-47
VP1635	outer membrane protein	2.24	2.50E-27
VP1642	trypsin	-2.50	9.48E-08

VP1656	translocator protein PopD	5.20	3.34E-88
VP1657	translocator protein PopB	5.96	5.33E-101
VP1659	hypothetical protein	4.67	9.51E-89
VP1660	type III secretion regulator	3.75	9.54E-35
VP1661	LcrR	3.97	2.43E-33
VP1662	low calcium response protein	4.01	1.99E-124
VP1663	YscY	4.03	4.98E-11
VP1664	type III secretion protein	3.80	9.48E-21
VP1665	type III secretion protein	2.12	1.39E-12
VP1666	hypothetical protein	2.24	1.46E-13
VP1667	outer membrane protein PopN	4.01	2.14E-102
VP1668	type III secretion system ATPase	3.31	1.01E-99
VP1669	type III secretion protein YscO	2.74	4.97E-10
VP1671	type III secretion system protein	4.88	7.87E-128
VP1672	type III secretion system protein	5.05	1.53E-74
VP1673	translocation protein in type III secretion	2.31	0.018181654
VP1674	translocation protein in type III secretion	3.38	1.84E-25
VP1675	translocation protein in type III secretion	3.29	2.96E-17
VP1679	hypothetical protein	-2.71	0.01258927
VP1680	hypothetical protein	5.09	3.08E-59
VP1682	hypothetical protein	4.65	5.05E-23
VP1683	hypothetical protein	5.01	3.82E-57
VP1684	hypothetical protein	3.59	3.28E-12
VP1685	hypothetical protein	-2.27	0.025406454
VP1686	adenosine monophosphate-protein transferase VopS	5.00	8.68E-70
VP1687	type III chaperone	4.06	3.11E-33
VP1688	type III secretion system protein	2.42	2.72E-15
VP1689	type III secretion protein	2.43	6.34E-17
VP1690	type III secretion lipoprotein	2.38	6.09E-25
VP1691	type III export protein	2.83	7.91E-10
VP1692	type III export protein	3.89	8.57E-73
VP1693	type III secretion protein	3.20	7.53E-31
VP1694	type III export protein YscF	3.97	9.01E-57
VP1695	type III export protein PscD	2.31	1.75E-43
VP1696	type III secretion protein YscC	2.66	2.37E-47
VP1698	hypothetical protein	2.44	1.72E-41
VP1702	hypothetical protein	4.37	1.40E-99
VP1707	hypothetical protein	-3.04	0.013715635
VP1708	6-phosphogluconate dehydrogenase	2.38	2.33E-30
VP1710	glucose-6-phosphate 1-dehydrogenase	2.26	1.78E-35
VP1718	cytochrome c551 peroxidase	3.47	3.07E-41
VP1719	aspartate kinase	-8.54	7.89E-280
VP1720	L-ectoine synthase	-9.32	7.52E-30
VP1721	diaminobutyrate--2-oxoglutarate aminotransferase	-9.84	9.26E-257

VP1722	L-2%2C4-diaminobutyric acid acetyltransferase	-9.18	1.84E-62
VP1726	glycine/betaine/proline ABC transporter	-7.06	2.47E-110
VP1727	glycine betaine/L-proline transport system permease	-7.80	1.21E-96
VP1728	glycine betaine transporter periplasmic subunit	-7.44	2.07E-150
VP1745	lipid A biosynthesis lauroyl acyltransferase	-2.06	0.001702427
VP1758	galactoside O-acetyltransferase	2.90	2.27E-16
VP1765	hypothetical protein	-2.83	2.79E-08
VP1767	hypothetical protein	-3.00	3.56E-05
VP1788	transposase	-2.48	0.000685663
VP1789	hypothetical protein	-2.38	0.000143697
VP1792	hypothetical protein	-2.06	2.75E-06
VP1793	hypothetical protein	-2.77	0.010128709
VP1798	hypothetical protein	-2.67	0.006468731
VP1799	hypothetical protein	-2.25	0.001332573
VP1800	hypothetical protein	-2.74	0.000178303
VP1801	hypothetical protein	-3.28	1.91E-13
VP1809	hypothetical protein	-2.77	2.61E-05
VP1810	hypothetical protein	-2.59	0.000297782
VP1813	hypothetical protein	-2.62	0.017342529
VP1819	hypothetical protein	-3.74	1.21E-06
VP1820	hypothetical protein	-2.47	0.001111497
VP1821	YefM protein	-2.61	2.74E-07
VP1834	hypothetical protein	-2.37	0.039941143
VP1836	hypothetical protein	-2.02	0.027453342
VP1840	hypothetical protein	-2.07	0.003849369
VP1841	hypothetical protein	-3.28	0.000277173
VP1847	hypothetical protein	-2.45	1.31E-05
VP1849	hypothetical protein	-2.89	9.04E-05
VP1872	hypothetical protein	-2.52	0.023546031
VP1880	L-serine dehydratase 1	2.38	6.63E-29
VP1881	hypothetical protein	-4.87	4.91E-51
VP1882	hypothetical protein	-2.59	5.55E-10
VP1883	6-pyruvoyl tetrahydrobiopterin synthase	-2.27	4.73E-13
VP1884	hypothetical protein	-4.18	0.000188912
VP1886	hypothetical protein	-2.91	0.019563163
VP1887	hypothetical protein	-2.59	0.042638297
VP1892	methyl-accepting chemotaxis protein	-2.03	4.27E-07
VP1894	hypothetical protein	-2.32	0.043730679
VP1900	aromatic amino acid aminotransferase	3.41	2.12E-52
VP1905	BCCT family transporter	-6.61	1.76E-94
VP1912	hypothetical protein	-4.55	2.81E-05
VP1924	thiol:disulfide interchange protein DsbE	-2.36	0.03804611
VP1928	cytochrome c nitrite reductase pentaheme subunit	-3.24	4.22E-16
VP1940	carboxynorspermidine decarboxylase	2.03	7.87E-23

VP1941	carboxynorspermidine dehydrogenase	3.40	1.49E-77
VP1942	diaminobutyrate-pyruvate transaminase %26 L-2%2C4-diaminobutyrate decarboxylase	2.86	2.15E-54
VP1943	CDP-diacylglycerol--glycerol-3-phosphate 3-phosphatidyltransferase	-3.21	7.97E-39
VP1952	hypothetical protein	4.98	3.52E-132
VP1955	Trp operon leader peptide	-5.07	6.51E-15
VP1966	proton/glutamate symporter	4.08	8.35E-83
VP1974	5-methyltetrahydropteroyltriglutamate--homocysteine S-methyltransferase	3.55	6.45E-10
VP1986	hypothetical protein	-2.01	0.008263891
VP1989	secretion protein	-2.96	0.000200103
VP1992	hypothetical protein	2.09	2.65E-17
VP1994	isochorismatase-like protein	4.71	1.91E-20
VP1995	ABC transporter ATP-binding protein	2.85	1.92E-63
VP1999	hypothetical protein	2.71	5.73E-36
VP2002	hypothetical protein	-2.20	0.004776136
VP2028	hypothetical protein	-2.78	3.69E-22
VP2042	hypothetical protein	-2.90	2.88E-07
VP2043	sodium-dependent transporter	-3.07	1.88E-32
VP2052	3-oxoacyl-ACP synthase	2.00	5.71E-24
VP2070	hypothetical protein	7.37	7.74E-39
VP2081	hypothetical protein	-2.19	8.25E-16
VP2082	acetate kinase	2.33	5.43E-41
VP2083	phosphate acetyltransferase	3.48	1.02E-80
VP2091	oligopeptide ABC transporter periplasmic oligopeptide-binding protein	2.12	5.91E-23
VP2110	hypothetical protein	2.66	1.26E-32
VP2121	bifunctional acetaldehyde-CoA/alcohol dehydrogenase	2.89	1.29E-42
VP2147	hypothetical protein	-2.04	0.000517089
VP2156	methionine sulfoxide reductase B	-2.41	7.57E-13
VP2157	glyceraldehyde-3-phosphate dehydrogenase	2.44	9.16E-31
VP2158	hypothetical protein	2.66	1.87E-33
VP2159	methyl-accepting chemotaxis transmembrane protein	2.64	1.64E-47
VP2164	hypothetical protein	-2.23	0.028326793
VP2165	hypothetical protein	-3.01	0.000137762
VP2169	hypothetical protein	-2.91	0.000144978
VP2171	proteinase inhibitor	-3.71	0.000335761
VP2174	regulatory protein	-2.25	0.029230625
VP2181	hypothetical protein	-3.18	0.009011558
VP2198	hypothetical protein	-2.67	1.96E-05
VP2213	long-chain fatty acid transport protein	-2.71	2.38E-09
VP2259	flagellin	3.22	5.57E-57
VP2280	Trp repressor-binding protein	-2.15	1.95E-08

VP2284	uracil phosphoribosyltransferase	3.13	4.65E-96
VP2285	phosphoribosylaminoimidazole synthetase	4.74	3.67E-164
VP2286	phosphoribosylglycinamide formyltransferase	2.29	7.83E-15
VP2313	phosphatidate cytidylyltransferase	-2.71	7.41E-15
VP2325	hypothetical protein	-3.34	6.76E-07
VP2326	acetyltransferase-like protein	2.22	1.60E-24
VP2330	hypothetical protein	-2.40	0.00014822
VP2337	sodium-dependent transporter	-2.19	3.77E-22
VP2366	GGDEF family protein	-2.37	0.00050205
VP2372	hypothetical protein	-5.83	2.32E-17
VP2380	2%2C3%2C4%2C5-tetrahydropyridine-2%2C6-dicarboxylate N-succinyltransferase	2.30	2.04E-37
VP2392	hypothetical protein	-3.97	0.000473934
VP2408	hypothetical protein	-2.59	0.04289025
VP2426	hypothetical protein	-4.10	0.000262412
VP2427	LysR family transcriptional regulator	2.32	1.53E-10
VP2437	NupC family protein	2.02	8.51E-24
VP2447	protease	2.28	9.60E-11
VP2448	protease	3.17	1.01E-28
VP2449	multidrug efflux pump VmrA	-2.43	5.90E-14
VP2450	MarR family transcriptional regulator	-2.31	1.27E-05
VP2467	outer membrane protein OmpU	4.13	1.06E-73
VP2474	iron-sulfur cluster insertion protein ErpA	-3.42	1.02E-27
VP2487	N%2CN'-diacetylchitobiose phosphorylase	2.17	7.97E-40
VP2491	Iron (III) ABC transporter periplasmic iron-compound-binding protein	2.57	5.43E-54
VP2492	ammonium transporter	5.09	2.91E-149
VP2493	nitrogen regulatory protein P-II	3.41	7.41E-05
VP2494	hypothetical protein	-4.62	2.01E-05
VP2500	DnaK suppressor protein	-2.47	4.25E-12
VP2507	pantoate--beta-alanine ligase	2.19	1.83E-41
VP2509	lipid A biosynthesis lauroyl acyltransferase	3.16	1.78E-33
VP2514	carbonic anhydrase	2.58	1.29E-47
VP2546	carbon storage regulator	-2.54	2.78E-11
VP2550	recombinase A	2.21	3.68E-40
VP2553	RNA polymerase sigma factor RpoS	2.57	8.82E-35
VP2560	cell division protein FtsB	-2.18	3.42E-05
VP2561	phosphopyruvate hydratase	3.63	2.60E-71
VP2581	hypothetical protein	-3.15	4.77E-10
VP2582	hypothetical protein	-2.10	7.35E-07
VP2583	hypothetical protein	2.56	8.34E-51
VP2585	hypothetical protein	-2.09	0.001872532
VP2589	hypothetical protein	-2.74	0.029936627
VP2593	D-3-phosphoglycerate dehydrogenase	2.90	1.63E-41

VP2596	LysE/YggA family protein	-5.27	5.17E-10
VP2599	fructose-bisphosphate aldolase	3.62	1.94E-76
VP2600	phosphoglycerate kinase	3.13	9.78E-87
VP2602	enterobactin receptor protein	2.27	4.42E-28
VP2604	hypothetical protein	-2.85	0.023336345
VP2647	hypothetical protein	-2.14	7.23E-12
VP2653	ornithine carbamoyltransferase	2.08	3.67E-11
VP2655	aspartate carbamoyltransferase	3.29	3.64E-38
VP2681	manganese transporter	2.01	1.92E-23
VP2711	UTP-glucose-1-phosphate uridylyltransferase	2.35	1.17E-50
VP2715	aspartate kinase	5.54	1.54E-250
VP2725	phage shock protein G	-3.88	4.91E-07
VP2730	hypothetical protein	-2.21	1.00E-10
VP2731	glucose-6-phosphate isomerase	2.70	9.25E-55
VP2736	hypothetical protein	-2.48	0.000914086
VP2755	hypothetical protein	-3.10	0.011450568
VP2757	argininosuccinate synthase	3.44	3.28E-47
VP2758	acetylglutamate kinase	3.35	8.72E-38
VP2763	5%2C10-methylenetetrahydrofolate reductase	2.19	1.61E-14
VP2766	transcriptional repressor protein MetJ	-2.09	0.006969228
VP2785	glutathione-regulated potassium-efflux system protein KefB	-2.70	4.21E-27
VP2788	ABC transporter ATP-binding protein	2.18	3.89E-48
VP2793	cAMP-regulatory protein	2.36	3.04E-40
VP2827	methyl-accepting chemotaxis protein	3.40	7.95E-94
VP2829	2%2C3-bisphosphoglycerate-independent phosphoglycerate mutase	3.65	2.60E-75
VP2835	hypothetical protein	4.09	3.49E-96
VP2837	hypothetical protein	-2.00	8.76E-05
VP2849	hypothetical protein	-3.41	1.84E-05
VP2880	acetyl-CoA carboxylase biotin carboxyl carrier protein subunit	2.69	2.36E-40
VP2881	acetyl-CoA carboxylase biotin carboxylase subunit	2.60	1.01E-30
VP2885	DNA-binding protein Fis	-3.44	0.000266293
VP2889	hypothetical protein	-2.18	0.006728023
VP2893	DNA-binding transcriptional activator CadC	-2.04	1.50E-23
VP2896	bifunctional phosphoribosylaminoimidazolecarboxamide formyltransferase/IMP cyclohydrolase	2.44	1.35E-29
VP2898	phosphoribosylamine--glycine ligase	2.58	2.70E-27
VP2913	hypothetical protein	-3.45	1.06E-07
VP2935	RNA-binding protein	-4.37	2.75E-18
VP2962	hypothetical protein	-2.88	0.006891174
VP3018	hypothetical protein	-3.41	3.58E-09
VP3021	aminopeptidase	2.26	3.99E-39
VP3028	camphor resistance protein CrcB	-3.30	1.30E-10
VP3036	5-(carboxyamino)imidazole ribonucleotide mutase	3.32	1.68E-77

VP3037	5-(carboxyamino)imidazole ribonucleotide synthase	2.63	1.68E-43
VP3050	hypothetical protein	-2.31	1.13E-35
VP3057	ComM-like protein	2.18	6.09E-22
VP3058	acetolactate synthase 2 catalytic subunit	3.29	1.74E-62
VP3059	acetolactate synthase 2 regulatory subunit	2.90	2.32E-41
VP3060	branched-chain amino acid aminotransferase	3.06	1.76E-54
VP3061	dihydroxy-acid dehydratase	3.47	1.54E-70
VP3062	threonine dehydratase	2.51	2.54E-41
VP3064	inner membrane transport protein YdhC	-2.33	4.32E-21
VP3066	amino acid ABC transporter permease	-2.17	0.000907877
VPA0029	hypothetical protein	-2.02	0.033124234
VPA0030	hypothetical protein	-3.26	0.00032098
VPA0031	sodium/glutamate symporter	-2.20	8.99E-39
VPA0044	hypothetical protein	-2.16	0.006786266
VPA0053	transcriptional regulator	-2.01	0.00038869
VPA0061	hypothetical protein	-3.43	2.76E-06
VPA0069	hypothetical protein	-2.44	0.013387023
VPA0126	acetyltransferase	-2.22	9.33E-10
VPA0128	biotin sulfoxide reductase	4.06	1.13E-96
VPA0131	hydroxyethylthiazole kinase	2.46	6.81E-37
VPA0132	transcriptional activator	2.61	7.38E-35
VPA0133	ABC transporter substrate-binding protein	3.01	4.83E-53
VPA0136	phosphomethylpyrimidine kinase	2.07	3.60E-29
VPA0146	glycerophosphoryl diester phosphodiesterase	2.05	5.26E-24
VPA0147	lactate dehydrogenase	3.15	5.18E-43
VPA0150	ferrichrome-iron receptor	-2.25	6.56E-37
VPA0168	multidrug resistance protein D	-3.46	2.90E-05
VPA0172	hypothetical protein	-2.38	0.000935622
VPA0173	ribosomal protein S6 modification protein	-2.88	0.00016064
VPA0183	C4-dicarboxylate transport transcriptional regulatory protein	-2.20	9.93E-30
VPA0200	hypothetical protein	-2.77	6.64E-05
VPA0213	hypothetical protein	-2.25	6.96E-05
VPA0234	4-aminobutyrate aminotransferase	2.02	0.00316461
VPA0243	virK protein	-2.83	3.53E-16
VPA0248	outer membrane protein OmpA	3.87	3.42E-38
VPA0265	flagellar basal body rod protein FlgC	2.59	0.001022493
VPA0267	flagellar hook protein FlgE	2.17	1.53E-10
VPA0288	hypothetical protein	-2.13	1.40E-07
VPA0289	hypothetical protein	-2.74	0.011817712
VPA0293	transcriptional regulator	-3.22	8.33E-14
VPA0296	oxidoreductase protein	-2.73	2.26E-15
VPA0301	methionine sulfoxide reductase A	2.40	5.30E-11
VPA0310	hypothetical protein	2.39	0.000443587
VPA0311	hypothetical protein	-3.01	0.004278832

VPA0312	hypothetical protein	-2.72	0.000201172
VPA0313	hypothetical protein	-2.00	2.17E-10
VPA0316	outer membrane protein	-2.34	0.002398336
VPA0336	hypothetical protein	-2.35	5.19E-07
VPA0345	hypothetical protein	-2.82	1.74E-06
VPA0346	hypothetical protein	-3.64	0.000239839
VPA0347	hypothetical protein	-2.65	5.09E-09
VPA0348	hypothetical protein	-2.80	5.55E-06
VPA0356	glycine betaine transporter	-4.90	2.33E-41
VPA0361	hypothetical protein	-4.79	1.13E-07
VPA0362	outer membrane protein	-2.07	1.45E-16
VPA0363	efflux protein	-3.39	2.08E-05
VPA0364	efflux protein	-4.43	1.69E-07
VPA0375	adenylosuccinate synthetase	3.90	2.16E-119
VPA0396	outer membrane lipoprotein	-2.16	3.36E-05
VPA0409	plasma membrane protein involved in salt tolerance	-3.31	1.31E-06
VPA0415	hypothetical protein	-2.04	0.015561751
VPA0418	hypothetical protein	-2.15	8.77E-19
VPA0426	TonB-like protein	2.34	7.42E-26
VPA0434	site-specific recombinase	-2.09	1.14E-11
VPA0450	hypothetical protein	4.82	1.86E-18
VPA0451	hypothetical protein	3.87	8.81E-09
VPA0454	DNA-binding stress protein	2.22	2.51E-05
VPA0459	collagenase	-2.24	1.64E-08
VPA0460	PAS factor	-2.25	1.92E-26
VPA0475	hypothetical protein	-3.56	5.93E-05
VPA0495	AraC family transcriptional regulator	-2.28	1.24E-08
VPA0521	hypothetical protein	-2.58	2.68E-05
VPA0523	hypothetical protein	-2.97	9.57E-08
VPA0527	outer membrane protein N	2.42	1.09E-29
VPA0528	hypothetical protein	-2.84	6.66E-08
VPA0534	hypothetical protein	-2.15	0.038876222
VPA0553	hypothetical protein	-2.32	0.00169589
VPA0566	alcohol dehydrogenase	-2.12	6.27E-46
VPA0567	amidotransferase domain-containing isoprenoid biosynthesis protein	3.39	8.94E-44
VPA0632	hypothetical protein	-3.16	0.002244517
VPA0641	LysR family transcriptional regulator	2.55	1.74E-22
VPA0642	glutathione S-transferase	2.34	1.77E-31
VPA0655	hypothetical protein	-4.29	1.19E-07
VPA0657	iron(III) ABC transporter periplasmic iron-compound-binding protein	2.51	2.03E-68
VPA0664	Fe-regulated protein B	2.09	5.31E-28
VPA0666	hypothetical protein	-2.47	1.38E-09

VPA0667	hypothetical protein	-4.49	4.21E-19
VPA0676	hypothetical protein	-2.82	0.009726206
VPA0678	hypothetical protein	-2.76	3.35E-11
VPA0691	riboflavin deaminase	-2.19	0.012342144
VPA0703	hypothetical protein	-2.89	2.01E-27
VPA0706	C4-dicarboxylate transporter DcuC	4.74	7.83E-154
VPA0707	delta-9 fatty acid desaturase	-2.37	2.14E-11
VPA0710	sensor histidine kinase/response regulator LuxN	-2.16	6.01E-19
VPA0746	chemotaxis protein CheV	2.15	1.15E-46
VPA0762	2-oxoglutarate/malate translocator	2.85	3.29E-12
VPA0775	hypothetical protein	-2.23	2.64E-07
VPA0776	mazG-like protein	-2.97	0.000378893
VPA0777	hypothetical protein	2.15	3.66E-18
VPA0789	hypothetical protein	-2.17	4.83E-06
VPA0791	hypothetical protein	-2.31	0.023324138
VPA0796	L-allo-threonine aldolase	3.16	8.10E-61
VPA0797	hypothetical protein	2.45	1.64E-08
VPA0800	hypothetical protein	-2.77	5.59E-18
VPA0801	glycine dehydrogenase	2.33	5.46E-55
VPA0819	amino acid ABC transporter permease	-3.35	8.47E-10
VPA0821	amino acid ABC transporter permease	-2.93	2.73E-20
VPA0822	amino acid ABC transporter ATP-binding protein	-2.60	1.42E-05
VPA0833	glucose-1-phosphate adenylyltransferase	5.86	4.29E-30
VPA0843	hypothetical protein	-2.07	4.17E-07
VPA0849	cytochrome b561	-2.30	1.52E-10
VPA0857	hypothetical protein	-2.22	2.49E-14
VPA0861	hypothetical protein	2.56	1.58E-53
VPA0862	hypothetical protein	-2.52	8.38E-06
VPA0865	hypothetical protein	-2.56	9.47E-13
VPA0870	trans-2-enoyl-CoA reductase	2.43	2.50E-38
VPA0878	hypothetical protein	-2.24	4.19E-15
VPA0893	phage structural protein	2.99	4.05E-07
VPA0901	major phage capsid protein P2	3.02	4.50E-06
VPA0903	hypothetical protein	2.20	0.000969929
VPA0914	hypothetical protein	-3.08	4.94E-05
VPA0922	NAD(P) transhydrogenase subunit alpha	2.53	1.36E-40
VPA0924	hypothetical protein	-2.20	0.001915597
VPA0933	hypothetical protein	-3.23	1.82E-17
VPA0939	adhesin	-2.09	0.00371212
VPA0940	anaerobic ribonucleotide reductase-activating protein	-2.32	0.020706638
VPA0950	hypothetical protein	-4.49	1.05E-06
VPA0960	hypothetical protein	-4.73	1.53E-07
VPA0963	sugar phosphate antiporter	-2.55	0.001639057
VPA0964	response regulator	-2.02	0.004000298

VPA0968	3-hydroxy-3-methylglutaryl-CoA reductase	-2.14	4.23E-06
VPA0987	nitrite reductase large subunit	2.16	0.000429358
VPA0988	nucleoside diphosphate kinase regulator	-2.40	1.28E-05
VPA0991	hypothetical protein	-2.34	2.77E-10
VPA1000	methyl-accepting chemotaxis protein	-2.53	6.29E-48
VPA1001	alanine racemase	-2.39	7.66E-07
VPA1005	D-lactate dehydrogenase	2.18	4.45E-42
VPA1011	L-allo-threonine aldolase	2.42	4.10E-42
VPA1017	AraC family transcriptional regulator	-2.12	2.65E-06
VPA1021	hypothetical protein	-2.60	0.008782251
VPA1032	hypothetical protein	-2.17	0.033553506
VPA1045	hypothetical protein	-2.40	1.30E-14
VPA1067	serine transporter	-6.56	3.88E-16
VPA1068	hypothetical protein	-2.54	1.82E-07
VPA1090	hypothetical protein	3.85	0.000963396
VPA1092	hypothetical protein	-2.02	5.06E-05
VPA1093	hypothetical protein	-2.76	0.001061211
VPA1094	hypothetical protein	2.47	2.22E-56
VPA1107	hypothetical protein	-3.04	2.46E-08
VPA1108	NAD(P)H-flavin reductase	2.40	3.31E-05
VPA1120	enoyl-CoA hydratase	2.06	0.005157805
VPA1127	enoyl-CoA hydratase/isomerase	2.84	5.88E-08
VPA1169	GTP cyclohydrolase I	2.54	1.88E-35
VPA1174	hypothetical protein	-3.23	0.001794907
VPA1186	outer membrane protein OmpA	-2.24	2.02E-24
VPA1208	preprotein translocase subunit SecF	3.51	1.49E-92
VPA1209	preprotein translocase subunit SecD	3.58	1.53E-82
VPA1241	hypothetical protein	-3.05	0.003790412
VPA1242	cytosine permease	2.06	2.52E-09
VPA1243	cytosine deaminase	2.59	8.44E-38
VPA1244	hypothetical protein	-3.64	0.00190406
VPA1246	hypothetical protein	-2.50	0.012733652
VPA1248	hypothetical protein	-3.10	0.011450568
VPA1255	hypothetical protein	-2.03	5.22E-11
VPA1256	hypothetical protein	-3.01	5.98E-23
VPA1257	hypothetical protein	-2.57	0.045647756
VPA1273	hypothetical protein	-2.26	0.017317245
VPA1283	MFS transporter	-2.14	0.013266557
VPA1284	hypothetical protein	-3.32	3.48E-23
VPA1290	hypothetical protein	-2.40	1.53E-07
VPA1305	hypothetical protein	-2.62	0.000468674
VPA1306	hypothetical protein	-3.10	2.41E-05
VPA1307	adhesion protein	-2.45	0.000102579
VPA1308	hypothetical protein	-2.04	7.52E-12

VPA1315	hypothetical protein	-2.13	0.000514047
VPA1332	transcriptional regulator ToxR	-2.23	1.83E-08
VPA1333	hypothetical protein	-3.23	0.001695144
VPA1356	hypothetical protein	-2.92	0.00070578
VPA1383	hypothetical protein	-2.87	0.022388054
VPA1384	hypothetical protein	-2.96	0.000371587
VPA1385	integral membrane protein	-2.98	0.001302919
VPA1398	hypothetical protein	-2.51	2.21E-13
VPA1403	capsular polysaccharide biosynthesis glycosyltransferase	2.69	1.42E-50
VPA1404	hypothetical protein	3.05	2.82E-74
VPA1405	polysaccharide export-like protein	2.24	3.44E-12
VPA1406	exopolysaccharide biosynthesis protein	2.88	1.78E-45
VPA1407	hypothetical protein	3.23	1.19E-57
VPA1408	lipopolysaccharide biosynthesis protein	2.86	5.49E-37
VPA1410	hypothetical protein	3.05	1.67E-47
VPA1411	glycosyltransferase	2.89	1.58E-45
VPA1416	oxidoreductase	3.05	1.43E-21
VPA1418	catalase	3.21	5.03E-68
VPA1424	PTS system fructose-specific transporter subunit IIABC	3.35	7.13E-40
VPA1425	mannose-6-phosphate isomerase	4.03	4.17E-30
VPA1443	protein secretion protein	2.28	3.83E-45
VPA1444	transport protein	2.10	2.50E-19
VPA1445	secreted calcium-binding protein	3.44	1.28E-43
VPA1448	hypothetical protein	-2.03	0.000339724
VPA1460	phosphate ABC transporter permease	-2.07	0.020038723
VPA1461	phosphate ABC transporter periplasmic phosphate-binding protein	-2.16	0.012653592
VPA1462	methyl-accepting chemotaxis protein	3.51	8.45E-30
VPA1479	hypothetical protein	-2.47	0.000120572
VPA1499	L-lactate dehydrogenase	2.18	8.06E-16
VPA1506	CsuB protein	-2.85	0.007834902
VPA1507	CsuA protein	-2.39	0.000485476
VPA1509	L-threonine 3-dehydrogenase	-3.51	2.98E-09
VPA1510	2-amino-3-ketobutyrate CoA ligase	-3.71	5.17E-10
VPA1524	hypothetical protein	-3.11	2.01E-30
VPA1525	hypothetical protein	-4.96	2.18E-06
VPA1542	flagellar biosynthesis protein FliP	-2.31	0.005131709
VPA1555	flagellar-specific transcription initiation factor sigma LafS	2.08	1.46E-08
VPA1570	hypothetical protein	-4.31	3.35E-11
VPA1584	hypothetical protein	2.13	8.46E-21
VPA1585	hypothetical protein	-3.76	1.05E-05
VPA1586	ferredoxin-NADP reductase	-3.09	5.83E-36
VPA1600	hypothetical protein	-2.16	0.00057311
VPA1610	hypothetical protein	-2.35	0.003274686

VPA1621	hypothetical protein	-2.84	0.023062105
VPA1623	transcriptional regulator MalT	2.40	2.21E-46
VPA1635	ornithine decarboxylase	3.07	2.65E-15
VPA1643	maltose operon periplasmic protein	2.05	5.19E-39
VPA1679	hypothetical protein	-2.21	0.0044981
VPA1681	organic hydroperoxide resistance protein	-2.27	2.25E-13
VPA1682	MarR family transcriptional regulator	-2.42	0.001022493
VPA1683	alkyl hydroperoxide reductase	2.40	1.16E-17
VPA1695	6-phospho-beta-glucosidase	2.17	2.84E-19
VPA1702	dicarboxylate-binding periplasmic protein	-2.39	0.008463216
VPA1703	small integral C4-dicarboxylate membrane transport protein	-2.31	0.005476277
VPA1708	keto-hydroxyglutarate-aldolase/keto-deoxy-phosphogluconate aldolase	2.28	1.05E-12
VPA1710	hypothetical protein	-2.92	0.019280074
VPA1714	hypothetical protein	-2.74	1.41E-10
VPA1722	hypothetical protein	-3.37	0.000160295
VPA1723	ribosomal protein N-acetyltransferase	2.13	6.30E-11
VPA1744	PTS system N-acetylmuramic acid transporter subunits EIIBC	-2.80	0.00237113
